# Effects of an intervention to prevent the bullying in first-grade secondary schools of Palermo, Italy: the BIAS study

**DOI:** 10.1186/s13052-019-0649-3

**Published:** 2019-05-27

**Authors:** Claudio Costantino, Alessandra Casuccio, Claudia Marotta, Stefania Enza Bono, Gianmarco Ventura, Walter Mazzucco, Francesco Vitale, Vincenzo Restivo, and the BIAS Study Working Group, Evelina Arcidiacono, Carlo Roberto Gambino, Maurizio Gentile, Sara Palmeri, Giovanna Ripoli, Claudia Emilia Sannasardo, Pierfrancesco Sannasardo, Francesco Scarpitta, Carlotta Vella

**Affiliations:** Department of Health Promotion Sciences, Maternal and Infant Care, Internal Medicine and Medical Specialties, University of Palermo, Via del Vespro n 133, 90127 Palermo, Italy

**Keywords:** Bullying, Prevention, Health promotion, Teachers, Students, Physical bullying, Verbal bullying, Indirect bullying

## Abstract

**Background:**

Bullying is one of the most common expressions of violence in the peer context during school years. This study investigates the prevalence of bullying and the short-term effects on students’ bullying perceptions of a preventive intervention conducted among teachers of first-grade secondary schools in Palermo, Sicily (Italy).

**Methods:**

Between the 2017/2018 and 2018/2019 school years, a pre-post intervention study was conducted among nine school institutions, sampled and categorized by neighbourhood socioeconomic index. A questionnaire investigating physical, verbal, and indirect bullying, the role of observers, prosociality, and resiliency in bullying was administered before and after intervention with formative cascade training of the teachers of the selected classes. Three different methods (sentinel questions, the five-question method, the ‘score of seven’ method) were used to detect the baseline level of bullying.

**Results:**

A total of 402 students participated in the study (72.7% response rate). A decrease in the number of bullying episodes after the intervention was reported by the students in all types of bullying explored (physical, verbal, and indirect bullying, observers, resiliency, and prosociality), with all three methods. In particular, a statistically significant decrease in all the bullying areas investigated (except for resiliency) was reported for students attending schools of an intermediate socioeconomic level.

**Conclusions:**

Even if many school-based interventions have been implemented to reduce school bullying throughout the world, this is one of the first conducted in Europe and it assesses the effectiveness among students of an anti-bullying intervention tailored for teachers. The encouraging results in reducing the number of bullying episodes together with the low cost in terms of human and economic resources could suggest an extension of this research on a regional/national scale.

## Background

Bullying affects the entire educational environment and has an impact on one’s right to a proper education. It represents the most common expression of violence in the peer context during school years [1]. Considering the strong amount of evidence of the negative health consequences for bullies, their victims, and observers as documented by studies from different countries, bullying in schools has become an important and complex global public health issue [1, 2].

According to a widely used research definition of bullying, a student is bullied or victimized when he or she is exposed repeatedly and over time to negative actions by one or more students [3]. Negative actions are further defined as when an individual (the perpetrator) intentionally inflicts or attempts to inflict injury or discomfort to another (the victim) [3]. Three criteria define aggressive behaviour as bullying: 1) repetition, 2) intentionality, and 3) an imbalance of power. Given these characteristics, bullying is often defined as the systematic abuse of power by peers [4]. Negative actions can be verbal (e.g. threats, taunts, teasing, name calling), physical (e.g. hitting, kicking, pushing, shoving, or pinching), and relational/social (e.g. social exclusion, rumour spreading), and can include the most recent forms of attacks on the Internet and using new technologies (also referred to as cyberbullying) [3, 5–8].

The effects of bullying and victimization have been widely studied during the past decades. Researchers have documented how bullying affects both the victims and the bullies, being associated with negative outcomes from all points of view, including poor academic performance and more school days missed for the victims and poor health outcomes, psychological maladjustment (e.g. in terms of well-being, self-esteem, and self-confidence), and psychosomatic health problems [2, 9–11]. This is why much effort has been exerted to contrast this phenomenon and, over the past 40 years, a large amount of research on anti-bullying has been produced [12–17].

Most of the research has examined school-based programmes, using a whole-school approach involving the individual students, parents, classrooms, and the entire school in one complex structure. Even if proven to been scientifically valid, the feasibility and capacity of the results of this research to fit within different contexts must be tested in different fields.

Both the selection and implementation of evidence-based school violence and bullying prevention and intervention programmes in schools have sometimes been problematic, perhaps because programmes are often chosen without consideration of the unique experiences and needs of each specific school or of the context in which the programme has proven to be successful [18, 19].

In Italy, bullying involves a significant percentage of school-age children: two in 10 children between the ages of 11 and 17 years report having been bullied two or more times in a month [20]. However, very little evidence on anti-bullying interventions implemented in Italy is available and none in Sicily, the first Italian region by territorial extension and the fourth by resident population [21, 22].

The Bullying in Sicilian Schools (BIAS) study was therefore designed to estimate the prevalence of the different forms of bullying observed or perceived by teachers and students in a representative sample of first-grade secondary schools in Palermo before and after the implementation of a bullying prevention intervention [23, 24].

## Materials and methods

A pre-post intervention study involving nine school institutions in the city of Palermo (Sicily) was conducted during the 2017/2018 and 2018/2019 school years. Approval of the Palermo Ethical Committee 1 (session of 12 July 2017, protocol no. 07/2017) was obtained.

### Study population

Nine school institutions were selected through cluster sampling based on sociodemographic criteria and then categorized into three levels – high (A), intermediate (B), and low (C) – of a neighbourhood socioeconomic index (SEI) based on the logarithm of the median household income, the proportion of adults aged 25 years or older with a high school diploma or college degree, and the proportion of people employed [23]. A total of 553 first-grade students in 30 classes were enrolled in the beginning of the study.

### Operating procedures and intervention

**Fig. 1:**
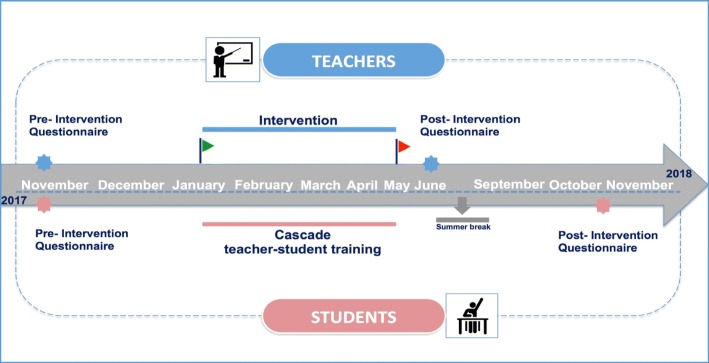
Timeline of the study

Figure 1 illustrates the time line of all the study’s activities. In October 2017, with the support of the Regional Bullying Observatory of Sicily, an operations meeting was organized for all the representatives of the schools enrolled in the study, as well as medical doctors and researchers from the Department of Health Promotion Sciences, Maternal and Infantile Care, Internal Medicine and Medical Specialities of the University of Palermo and healthcare professionals from the local health unit of Palermo, with proven experience in child and adolescent mental health.

Following a baseline assessment of teachers’ perceptions of the bullying in the selected classes through an online questionnaire administered in November 2017 [23], a specific intervention was administered to the teachers between January and May 2018, with training and informative sessions on topics closely related to bullying; this was followed by a specific intervention on the students administered by the trained teachers. Finally, in June 2018, a post-intervention questionnaire was administered with the same procedure as before, to detect any changes in the prevalence of bullying perceived by the teachers [24].

At the same time, in December 2017, the baseline prevalence of bullying was assessed from the students’ perspective. After obtaining written consent from their parents, the students were given a structured and previously validated questionnaire investigating the six main areas of practical interest and research on bullying: 1) physical bullying, 2) verbal bullying, 3) indirect bullying, 4) observers of bullying, 5) resiliency, and 6) prosociality [23].

To organize dedicated activities for the students and to address and prevent bullying at school, during the teachers’ intervention time frame (January to May 2018), cascade teacher–student training was given, where the teachers provided the content they learned to their students through participative methods, such as cooperative learning, peer education, and role playing. A post-intervention questionnaire was then administered during October and November 2018 to the students who had responded to the pre-intervention questionnaire and were currently attending the third class during the 2018/2019 school year. The aim was to note any short-term changes in the prevalence of bullying among students after the intervention was conducted on their teachers.

The questionnaires used to detect the prevalence of bullying for both the teachers and the students had been previously published together with a complete BIAS protocol study [23]. They were created by the BIAS research group based on an extensive literature review and on teachers’ experiences but had not been validated. In Table 1, we briefly review the student questionnaire and the three different methods (sentinel questions, the five-question method, and the ‘score of seven’ method) of analysing the prevalence of bullying [23].

**Table 1:** The student questionnaire

The questionnaire consisted of the following different sections:	
General information, that is, gender, age, nationality, school institution, and class attended;	
• Five questions about the area of verbal bullying (e.g. being called an offensive nickname);	
• Five questions about the area of physical bullying (e.g. being attacked);	
• Five questions about the area of indirect bullying (e.g. being ignored or secluded);	
• Five questions about the area of resilience (e.g. talking to someone about having been bullied);	
• Five questions about the role of observers (e.g. seeing a classmate being teased and not intervening);	
An open-ended final questionnaire section to freely express thoughts about 1) the content of the questionnaire and 2) bullying in general.	
The analysis was constructed as follows:	
For each answer, a score between one (never) and five (very often) was assigned. The score was then used to detect the baseline level of bullying with the following three methods:	
1. Sentinel questions, where the presence or absence of bullying was investigated through yes/no answers to the most significant questions in an area. The responses *very often*, *often*, and *occasionally* were considered affirmative answers.	
2. The five-question method, which considered bullying to be present whenever the student answers yes (i.e. *occasionally*, *often*, or *very often*) to at least one of the items in the survey area.	
3. The score of seven method, where the answers to each question were scored and added and the presence or absence of bullying was then determined, where the value of seven was considered the cutoff (i.e. the respondent could answer *occasionally* at least to at least one of the questions in an area).	
Both the pre- and post-intervention questionnaires were identical. The only the difference was that the pre-intervention questionnaire investigated an interval period of ‘the last three months’, whereas the reference time frame in the post-intervention questionnaire also included the previous six months, before the summer break.	

### Working group interventions

The formative activities were conducted and supervised by the research project’s five working groups (WGs). Each WG was composed of three members: one from the Sicilian Regional Bullying Observatory, one from the Palermo Local Health Unit, and one from the University of Palermo’s Department of Health Promotion. All the members of the class councils involved in the study participated in four meetings, each lasting five hours, organized by the WGs from January to May 2018. All meetings were organized in the school setting outside of school hours. During the meetings, the WGs met the teachers to discuss the data collected with the pre-intervention questionnaire and to develop and plan effective activities to increase bullying awareness and promote preventive actions in their school’s context. Participatory approaches to the planning (e.g. word café, role playing, goal-oriented project planning) were implemented to structure the activities to target the students. From March to May 2018, during the WGs’ formative activity, the teachers organized, with their students, initiatives planned through these participatory approaches to address and prevent bullying at school. In particular, the teachers organized these initiatives with their students during normal school activities, using at least one of the three following methods suggested by the WG members: cooperative learning, peer education, and/or role playing.

### Data collection and analysis

The questionnaires were self-administered by the students of the individual classes through the Google Forms online platform. The data obtained were then exported to a Microsoft Excel spreadsheet and analysed with the statistical software Epi Info (v. 7.2.2.6). Descriptive and comparative analyses were performed by school socioeconomic index. All categorical variables were reported as absolute and relative frequencies (percentages). Chi-squared tests (with Fisher’s correction where appropriate) were used to compare categorical variables.

## Results

Overall, 553 students enrolled in the 30 selected classes were invited to participate in the study. Of these, 151 (27.3%) were absent on one of the two days of the questionnaires’ administration. The pre- and post-intervention questionnaires were administered to the following groups: SEI school group A comprised three schools, for a total of 11 classes and 171 students (93 girls, or 54%; mean age 11.6); SEI school group B comprised four schools, for 13 classes and 180 students (97 girls, or 54%; mean age 12.0); and SEI school group C comprised two school institutions, accounting for six classes and 51 students (26 girls, or 51%; mean age 12.1). A total of 402 students thus participated in the study (response rate 72.7%), 210 (52.2%) of whom were female, with a mean age of 12.3 years, or 12 years and four months (standard deviation 1.3 years; median 12.5 years; range 11–16 years), by the end of the study, and seven (1.9%) were foreign. All teachers who carried out all or part of their teaching activities in the sampled classes were invited to participate in the project. With the aim of including all the teachers’ opinions and for them to determine together the answers to be provided, the questionnaire was completed collegially by the entire class council. All teachers chose to participate in the project. There were a total of 45 class councils. The mean age of the teaching staff was 57.2 years. 83% of the teachers were female.

The results of the survey in the six bullying areas were analysed using the three methods, that is, sentinel questions, the five-question method, and the score of seven method. The prevalence of different types of bullying (physical, verbal, indirect) decreased after the intervention, with the percentage changes varying according to the estimation method used (Table 2).

**Table 2:** Prevalence of different types of bullying in pre- and post-intervention, and percentage changes, among the 402 students of the nine institutions enrolled in the BIAS study

Area	*Method*	Pre n (%)	Post n (%)	% difference
Physical bullying	*Sentinel question*	13 (3.2)	6 (1.5)	- 1.7
	*Five-question*	103 (25.6)	86 (21.4)	- 4.2
	*Score of seven*	141 (31.5)	118 (29.4)	- 2.1
Verbal bullying	*Sentinel question*	55 (13.7)	44 (10.9)	- 2.8
	*Five-question*	150 (37.3)	128 (31.8)	- 5.5
	*Score of seven*	228 (56.7)	204 (50.7)	- 6
Indirect bullying	*Sentinel question*	41 (10.2)	36 (9)	- 1.2
	*Five-question*	118 (29.4)	113 (28.1)	- 1.3
	*Score of seven*	174 (43.3)	156 (38.8)	- 4.5
Observers	*Sentinel question*	54 (13.4)	30 (7.5)	- 5.9
	*Five-question*	106 (26.4)	82 (20.4)	- 6
	*Score of seven*	163 (40.5)	124 (30.8)	- 9.7
Resiliency	*Sentinel question*	120 (29.9)	134 (33.3)	+ 3.4
	*Five-question*	192 (47.8)	178 (44.3)	- 3.5
	*Score of seven*	246 (61.2)	223 (55.5)	- 5.7
Prosociality	*Sentinel question*	44 (10.9)	15 (3.7)	- 7.2
	*Five-question*	313 (77.9)	385 (70.9)	- 7
	*Score of seven*	347 (86.3)	325 (80.8)	- 5.5

Specifically, the prevalence of physical bullying decreased when estimated with the sentinel question method (− 1.7% change), the five-question method (− 4.2% change), and the score of seven method (− 2.1% change). The prevalence of verbal bullying decreased between the pre- and the post-intervention questionnaires with all three methods (− 2.8, − 5.5%, and − 6.0% changes respectively).

The prevalence of indirect bullying was slightly reduced as measured by both the sentinel question method (− 1.2%) and the five-question method (− 1.3%) and decreased from 43.3 to 38.8%, for a change of − 4.5%, using the score of seven method between the pre- and post-intervention questionnaires.

The data on observers also shows a decrease after the intervention: from 13.4 to 7.5% with the sentinel question method, from 26.4 to 20.4% with the five-question method, and from 40.5 to 30.8% with the score of seven method. A reduction was also reported in the prosociality area with all three methods (− 7.2, − 7%, − 5.5%, respectively). Finally, the prevalence of resiliency increased with the sentinel question method (+ 3.4%) but decreased with both the five- question method (− 3.5%) and the score of seven method (− 5.7%).

Table 3 shows the results for each bullying area before and after the intervention by the neighbourhood SEI of the nine first-grade secondary schools. A statistically significant change between the pre- and post-intervention results was reported for the students attending schools with SEI level B (intermediate level) with regard to physical, verbal, and indirect bullying according to all the three methods. The reduction of indirect bullying in intermediate-SEI schools using the five-question method (from 28.6 to 23.2%; *p*-value 0.23) was the only result that was nonsignificant.

**Table 3:** Prevalence of different types of bullying with confidence interval (95%), among the 402 students enrolled in the pre and post intervention, according to the neighborhood socio-economic index (SEI) of the nine institutions

Method	School SEI	Physical			Verbal			Indirect		
		Pre n (%; CI 95%)	Post n (%; CI 95%)	*p*-value	Pre n (%; CI 95%)	Post n (%;CI 95%)	*p*-value	Pre n (%;CI 95%)	Post n (%;CI 95%)	*p*-value
Sentinel question	A	3 (1.8; -0.2 – 3.8)	3 (1.8; -3.2 – 6.8)	0.98	19 (11.1; 6.3-15.9)	21 (12.6; 7.6-17.6)	0.68	15 (8.8; 4.5-13.1 )	24 (14.4; 9.1-19.7)	0.11
	B	8 (4.4; 1.5-7.3)	2 (1.1; -0.4-2.6)	**<0.05**	26 (14.3; 9.4-19.2)	15 (7.9; 4.1-11.7)	**<0.05**	16 (8.8; 4.8-12.8)	7 (3.7; 1-6.4)	**<0.05**
	C	2 (3.9; -7.8-15.6)	1 (2.2; -2.2-6.6)	0.63	10 (19.6; 7.9-31.3)	8 (17.8; 6.5-29.5)	0.82	10 (19.6; 7.9-31.3)	5 (11.1; 1.8 - 20.4)	0.25
Five-question	A	33 (19.5; 13.5-25.5)	39 (23.4; 17-29.8)	0.39	55 (32.5; 25.4 - 39.6)	60 (35.9; 28.6-43.2)	0.51	41 (24.3;17.8-30.8)	53 (31.7;24.7-38.7)	0.13
	B	54 (29.7; 22.9-36.2)	33 (17.4; 12-22.8)	**<0.01**	67 (36.8; 29.9-43.7)	49 (25.8; 19.6-32)	**<0.05**	52 (28.6; 22.2-35)	44 (23.2; 17.2-29.2)	0.23
	C	16 (31.4; 16.7-46.1)	14 (29.5; 15.8-43.2)	0.86	28 (54.9; 40.2-69.6)	19 (42.2; 27.6-56.8)	0.22	25 (49; 34.2-63.8 )	16 (35.6; 21.5-49.7)	0.18
Score of seven	A	45 (26.6; 19.9-33.3)	54 (32.3; 25.2-39.4)	0.25	85 (50.3; 42.7-57.9)	94 (56.3; 48.8-63.8)	0.27	58 (34.3;27.1-41.5)	69 (41.3; 33.8-48.8)	0.18
	B	74 (40.7; 33.7-47.7)	46 (24.2; 18.1-30.3)	**<0.001**	104 (57.1; 50-64.2)	82 (43.2; 36.1-50.3 )	**<0.01**	86 (47.3; 40.2-54.4)	63 (33.2; 26.5-39.9)	**<0.01**
	C	22 (43.1; 30.6-55.6)	18 (40; 25.5-54.5)	0.76	39 (76.5; 64-89)	28 (62.2; 47.9-76.5)	0.13	30 (58.8; 44.3-73.3)	24 (53.3; 38.6-68)	0.59
Method	School SEI	Prosociality			Observers			Resiliency		
		Pre n (%; CI 95%)	Post n (%; CI 95%)	*p*-value	Pre n (%; CI 95%)	Post n (%;CI 95%)	*p*-value	Pre n (%;CI 95%)	Post n (%;CI 95%)	*p*-value
Sentinel question	A	12 (7.1; 3.2-10.3)	7 (4.2; 1.2-7.2)	0.25	16 (9.5; 5.1-13.9)	11 (6.6; 2.9-10.3)	0.33	46 (27.2; 20.4-34)	51 (30.5; 23.5-37)	0.50
	B	24 (13.2; 8.4-18)	5 (2.6; 0.3-4.9)	**<0.05**	28 (15.4; 10.3-20.5)	12 (6.3;2.8-9.8)	**<0.01**	58 (31.2; 24.6-37.8)	65 (34.2; 27.4-41)	0.63
	C	10 (19.6; 8.9-30.3)	5 (11.1; 3.7-18.5)	0.26	10 (19.6; 7.9-31.3)	7 (15.6; 4.9-26.3)	0.61	16 (31.4; 17.7-45.1)	18 (40;25.5-54.5)	0.38
Five-question	A	128 (75.7; 69.2-82.2)	128 (76.7; 70.3-83.1)	0.85	31 (18.3; 12.4-24.2)	26 (15.6; 10.1-21.1	0.50	75 (44.4;36.8-52)	75 (44.9; 37.4-52.4)	0.92
	B	139 (76.4; 71.8-81)	124 (65.3; 59.2-71.4)	**<0.05**	54 (29.7; 23.2-36.2)	38 (20;14.3-25.7	**<0.05**	90 (49.5;42.1-56.6)	80 (42.1; 35.1-49.1)	0.16
	C	46 (90.2; 81.4-99)	33 (73.3; 60.2-86.4)	0.06	21 (41.2; 26.7-55.7)	18 (40; 25.5-54.5)	0.90	27 (52.9; 38.2-67.6)	23 (51.1; 36.3-65.9)	0.86
Score of seven	A	142 (84; 78.4-89.6)	143 (85.6; 80.3-90.9)	0.68	48 (28.4;21.6-35.2)	46 (27.5;20.8-34.2)	0.86	94 (55.6;48.1-63.1)	89 (53.3;45.7-60.9)	0.67
	B	157 (86.3;81.4-91.2)	144 (75.8;69.7-81.9)	**< 0.01**	88 (48.4;41.3-55.5)	58 (30.5;23.9-37.1)	**<0.001**	116 (63.7;56.8-70.6)	106 (55.8;48.7-62.9)	0.12
	C	48 (94.1;87.2-87.2)	38 (84.4;73.7-95.1)	0.12	27 (52.9;38.2-67.6)	20 (44.4;29.7-59.1)	0.41	36 (70.6;57.2-84)	28 (62.2;47.9-76.5)	0.39

At time, the entries in boldface are all significant with three different level of significance observed: < 0.05 (5 times), < 0.01 (4 times), < 0.001 (1 time)

In the prosociality and observer areas, a statistically significant decrease was demonstrated for the students of schools with an intermediate SEI (level B) with all three methods. Moreover, an increase in all types of bullying among schools with a high SEI (level A) and a decrease among students of low-SEI schools (level C) was observed, neither of them statistically significant. Finally, for the resiliency area, none of the changes observed in any of the schools were significant.

## Discussion

This study aimed to investigate the short-term effects of bullying and bullying-related positive attitudes on students’ perceptions after a prevention intervention on teachers of a sample of nine first-grade secondary schools in Palermo.

Within the theoretical framework of the teacher–student training cascade effect, teachers were identified as the primary target of an anti-bullying intervention, subsequently translating strategies and methodologies for the prevention of bullying in the classroom [25–27]. To assess the short-term impact of this intervention, a pre-post intervention questionnaire was administered to 428 students and then analysed using three different methods – the sentinel question method, the five-question method, and the score of seven method – for each of the variables in the study [23].

A decrease in the number of bullying episodes between the pre- and post-intervention surveys was reported by the students in all areas of bullying explored (verbal, physical, and indirect) and was confirmed by each of the three estimation methods. In particular, verbal bullying demonstrated the most significant decrease, together with the physical bullying and observer areas, where the decrease documented also appears encouraging. Among the positive attitudes explored, only the effects for resilience increased with the sentinel question method, although the areas of prosociality and resiliency showed a decrease with the five-question and score of seven methods, unlike the other evidence [22]. These results can be partially explained by the fact that the reduction in the number of bullying episodes inevitably leads to a reduction in related positive attitudes (e.g. reporting the case to parents/teachers).

Unlike other studies estimating the prevalence of bullying with a single-item method (sentinel question), the present study provides not only different measures of bullying prevalence but also a more detailed analysis of the different types of bullying (physical, verbal, indirect, observer role) [12, 21, 22]. Given an analysis of the changes between the pre- and post-intervention questionnaire results according to the neighbourhood SEI of the schools enrolled, a statistically significant reduction in the prevalence in all areas of bullying was observed among students attending schools with an intermediate SEI.

This evidence could suggest different degrees of effectiveness of the intervention conducted in each class by the teachers, who were left free to adapt the content acquired during the formative intervention with their students according to the context’s specificities. The use of a non-prestructured intervention for the students but with a focus on the class could also have been important, given specific aspects of Sicilian culture (e.g. violence, especially in the context of neighbourhood disadvantages) within the genesis of bullying in these specific settings.

In line with method suggested by Ttofi MM et al., the WGs of experts and researchers from various disciplines (education, mental health, public health) that conducted the anti-bullying interventions successfully contributed in reducing the prevalence of bullying [12].

Some limitations of the present study should be considered. First, the study only evaluated students’ self-reported perceptions of bullying, which could have biased the results. Even if this aspect could be partly overcome, future official bullying reports should consider integrating these results with the teachers’ perspective, for a wider interpretation of the phenomenon, especially in the future, when laws will require teachers to officially report every episode of bullying within the school context [28]. The students’ results differed from the teachers’ particularly in the areas of physical bullying, where the teachers reported a zero prevalence in the post-intervention period [24]. This discrepancy highlights how teachers might not be aware of episodes of bullying in their absence or that occur in the school context but outside of the classroom (e.g. bathrooms, halls, outside areas).

Moreover, the school sample involved only urban settings, introducing a potential selection bias that could be avoided by extending future research to suburban and rural areas. On the other hand, the schools selected also represent very different socioeconomic backgrounds, an important aspect for the generalizability of the intervention. Finally, only short-term effects were evaluated and the effects will be difficult to follow up in the longer term, since this aspect was not considered in the study structure and no individual contacts for future communications were foreseen.

## Conclusions

In conclusion, even if many school-based interventions have been implemented to reduce bullying in school throughout the world, this is one of the first to be conducted in Europe that assesses the effectiveness among students of an anti-bullying intervention tailored for teachers. The encouraging results obtained in reducing the number of bullying episodes, together with the low cost in term of human and economic resources, could suggest extension of the research on a regional basis and to different school grades, to propose a common strategy to address one of the most important public health issues in school today. In particular, it would be interesting to note any differences between schools in more urbanized, metropolitan areas and those in rural contexts in terms of the prevalence of bullying and the effectiveness of the proposed interventions. Lastly, integration of future research in the cyberbullying area could allow for the development of a combined preventive strategy [25, 29]. Additionally, parental involvement, already known to be effective [12], should be considered.

## Abbreviations

BIAS: Bullying In Sicilian Schools
SEI: Socioeconomic index
